# Diagnostic value of plasma heat shock protein 90α and inflammatory markers in prostate cancer

**DOI:** 10.3389/fonc.2025.1683060

**Published:** 2026-01-14

**Authors:** Weiwei Zhang, Yajun Miao, Lisheng Liu

**Affiliations:** 1 Department of Clinical Laboratory, Shandong Cancer Hospital and Institute, Shandong First Medical University and Shandong Academy of Medical Sciences, Jinan, Shandong, China; 2 Shandong Provincial Third Hospital, Cheeloo College of Medicine, Shandong University, Jinan, Shandong, China

**Keywords:** diagnostic value, inflammatory markers, plasma Hsp90α, prostate cancer, TNM stages

## Abstract

**Objective:**

To assess plasma levels of heat shock protein 90α (Hsp90α) and inflammatory markers, and evaluate their diagnostic potential in prostate cancer (PCa).

**Patients and methods:**

Patients were divided into two groups based on histopathological diagnosis: PCa group and benign prostatic disease group. The levels of plasma Hsp90α and inflammatory markers were compared between groups. Diagnostic performance was evaluated using receiver operating characteristic (ROC) curve analysis. Bioinformatics analysis (Gene Set Enrichment Analysis, GSEA) was further performed to explore the potential Hsp90α-related signaling pathways in PCa.

**Results:**

Plasma Hsp90α levels were significantly higher in PCa patients compared to benign prostatic disease patients (102.8 ± 89.77*vs.*62.57 ± 34.82 ng/mL, p < 0.001), while PLT (213 ± 58.95*vs.*266 ± 70.62 *10^9^/L, *p* < 0.05) and platelet-to-neutrophil ratio (PNR, 62.48 ± 24.01*vs.*74.33 ± 25.19, *p* < 0.05) were significantly lower. Plasma Hsp90α levels showed strong correlations with the M stage (*p* < 0.001), N stage (*p* < 0.01) and Clinical stage (*p* < 0.001), PNR was negatively correlated with M stage (*p* < 0.01), decreasing with tumor progression. ROC curve analysis revealed moderate diagnostic value for Hsp90α (AUC = 0.661), PLT (AUC = 0.601), and PNR (AUC = 0.590). GSEA indicated that significant correlation between Hsp90 levels and protein secretion-related pathways and cell cycle regulation signaling pathways.

**Conclusions:**

In summary, this study demonstrates the potential clinical utility of plasma Hsp90α as an auxiliary diagnostic biomarker for PCa, particularly in advanced or metastatic disease. Furthermore, we are the first to report the diagnostic and distant metastasis risk assessment potential of PNR in PCa. Notably, diagnostic models integrating Hsp90α and PNR with prostate-specific antigen (PSA) exhibited superior performance compared to PSA alone, suggesting their complementary role. Through integrated bioinformatics analyses, we have elucidated the molecular mechanisms by which Hsp90α drives PCa progression. These findings provide novel mechanistic insights into the pathophysiology of PCa and establish a foundation for developing future diagnostic strategies and targeted therapies focusing on Hsp90α or its associated pathways.

## 1 Introduction

Prostate cancer (PCa) is a malignant tumor arising from the uncontrolled proliferation of prostate epithelial cells (1). According to the global cancer statistics in 2022, PCa is a common cancer among men, ranking second with an incidence rate of 14.2%, and it ranks fifth in terms of the mortality rate, which is 7.3%, among all male cancer-related deaths (2). Recent data released by the American Cancer Society indicate that the estimated number of new PCa cases has surpassed that of lung cancer, now ranking first in incidence, while maintaining the second leading cause of cancer death among men. Moreover, its incidence has continued to rise in recent years, posing a substantial challenge to healthcare systems worldwide (3).

Heat shock protein 90 (Hsp90), which includes the isoforms Hsp90α and Hsp90β, is a molecular chaperone typically induced under cellular stress that facilitates the maturation of a wide range of client proteins (4). Hsp90α has emerged as a research focus due to its key roles in modulating signal transduction, especially in tumorigenesis. Clinical studies reported that the levels of plasma Hsp90α in lung cancer patients and liver cancer were significantly higher compared to healthy controls (5, 6). Evidence also suggests that Hsp90α may server as a prognostic indicator in triple-negative and/HER2+ breast cancer, which can increase the risk of recurrence and distant metastasis (7). Additional, extracellular Hsp90 (eHsp90) further promotes cancer progression by facilitating epithelial- mesenchymal transition (EMT) (8). Moreover, a recent study showed that Hsp90 level is positively associated with ovarian cancer mortality and is a potential prognostic indicator of ovarian cancer (9). As an ATP-dependent chaperone, Hsp90 is essential for the maturation, stabilization, and activation of diverse client proteins. It also participates in protein folding, complex assembly, and regulated degradation. Moreover, Hsp90 influences apoptotic pathways through the modulation of key factors such as p53, nuclear factor kappa-B (NF-κB), Akt (protein kinase B), and RAF-1 (10).

Tumor progression and metastasis involve not only malignant cells but also inflammatory responses (11). Related indicators of the inflammatory response, such as the neutrophil-lymphocyte ratio (NLR) and systemic immune-inflammation index (SII), have been widely used in the diagnosis and prognosis of cancer patients (12, 13). Numerous studies have reported on the relevance of inflammatory markers in the context of PCa (14, 15).

Although Hsp90α has demonstrated significant diagnostic and prognostic value across multiple cancer types, its utility—either alone or in combination with inflammatory markers—remains underexplored in PCa. In this study, we quantified plasma Hsp90α concentrations in peripheral blood samples from 144 patients with PCa and 64 with benign prostatic diseases. We subsequently analyzed the relationship between Hsp90α levels, inflammatory markers, and clinical-pathological characteristics. Furthermore, we employed bioinformatics approaches, including functional enrichment and gene set enrichment analysis (GSEA), to identify key biological pathways and metabolic processes regulated by Hsp90 in PCa.

## 2 Materials and methods

### 2.1 Patients

From January 2, 2019 to October 3, 2024, a total of 208 prostate disease patients were enrolled in this study in Shandong Cancer Hospital and Institute, affiliated to Shandong First Medical University. The cohort comprised 144 PCa patients, 64 patients with benign prostatic diseases (59 benign prostatic hyperplasia (BPH); 3 prostatitis; 2 prostatic cysts). Diagnosis were confirmed by histopathological examination of surgical specimens or biopsy results. PCa staging followed the 8th edition of the TNM classification system by the American Joint Committee on Cancer (AJCC). All patients were underwent prostate surgery/biopsy with assessment of clinical symptoms and signs, together with measurement of plasma Hsp90α levels, complete blood count (CBC), and serum prostate-specific antigen (PSA) levels. Exclusion criteria included prior antitumor therapy or surgical resection. Written informed consent was obtained from all participants. This study protocol was approved by the Local Ethics Committee of the Affiliated Shandong Cancer Hospital and Institute, and it was conducted in accordance with the Declaration of Helsinki and current hospital ethical guidelines.

### 2.2 Measurement of plasma Hsp90α levels

Venous blood specimens of patients were collected into an ethylenediaminetetraacetic acid (EDTA) anticoagulant tube. The level of Hsp90α was detected using a human Hsp90α ELISA kit (Yantai Protgen Biotechnology Development Co., Ltd., Yantai, China), according to the manufacturer’s instructions and a published protocol (16). Briefly, freshly collected blood specimens were centrifuged at 3000 revolutions per minute (rpm) for 10 minutes to separate the plasma layer. The standard solutions, quality controls and plasma specimens were diluted with the sample diluent in proportion and added to a 96-well microplate. Subsequently, 50 μL of the working solution of anti-Hsp90α antibody labeled with horseradish peroxidase (HRP) was added to each well, and the incubation was performed at 37 °C for 1 hour. by adding Tetramethylbenzidine (TMB), a chromogenic substrate, the enzymatic reaction was initiated, and finally, the absorbance value was measured at 450 nm using a microplate reader. Hsp90α concentrations were calculated using a standard curve generated from known standards.

### 2.3 Detection of tumor markers and CBC

Serum levels of PSA, testosterone and Carcinoembryonic antigen(CEA) were measured using the electrochemiluminescence immunoassay kit (Cobas, Roche Diagnostics, Germany) following the manufacturer’s instructions. CBC were counted using Flow cytometry and fluorescence staining technique (XN9000, Sysmex, Japan) according to the manufacturer’s instruction. Serum samples were obtained in tubes without anticoagulant, allowed to clot at room temperature for 30 minutes, and centrifuged at 3000 rpm for 10 min to separate the serum layer.

### 2.4 Expression pattern of HSP90AB1 in the cancer genome atlas

To investigate the expression pattern of HSP90AB1 in PCa across different clinical subgroups, RNA-seq data (FPKM-normalized) and corresponding clinical annotations for PCa patients were downloaded from TCGA via the TCGA biolinks R package (version 4.4.3) (17) [or “publicly available through the TCGA Data Portal”, or specify the exact platform/tool]. A total of 548 samples (496 tumor tissues and 52 adjacent normal tissues) were included in the analysis.

### 2.5 Bioinformatics analyses

First, RNA-sequence V2 RSEM data, which included 19,392 genes expressed in PCa tissues, were downloaded from https://www.cbioportal.org/by using the “cgdsr” R package. The top 100 genes positively correlated with HSP90AB1 level (Pearson’s correlation ≥ 0.3, P < 0.0001) were filtered by calculating Pearson’s correlation coefficients. Then, bioinformatics analyses of functional signalling pathways were performed via Metascape (18), an online platform (https://metascape.org/). Pathway and process enrichment analyses for each gene list were performed using the following ontology sources: KEGG Pathway, GO Biological Processes, Reactome Gene Sets, Canonical Pathways, Cell Type Signatures, CORUM (Protein Complex Database), TRRUST (Transcription Factor Regulatory Network Database), DisGeNET (Disease Gene Database), PaGenBase (Pan-Cancer Gene Database), Transcription Factor Targets and WikiPathways (9). A q-value<0.05 was used as the threshold for indicating significance.

### 2.6 GSEA analysis of Hsp90

Te gene expression profle of TCGA-PCa was obtained through TCGA, which can be accessed via this link: https://www.cancer.gov/ccg/research/genome-sequencing/tcga. For Gene Set Enrichment Analysis (GSEA) (19), the GSEA software (version 3.0) was obtained from the GSEA website (DOI: 10.1073/pnas.0506580102, http://software.broadinstitute.org/gsea/index.jsp). Samples were divided into two groups based on high and low levels of HSP90AB1. The c2.cp.kegg.v7.4.symbols.gmt subset was downloaded from the Molecular Signatures Database (20) (MSigDB; DOI: 10.1093/bioinformatics/btr260, http://www.gsea-msigdb.org/gsea/downloads.jsp) to evaluate relevant pathways and molecular mechanisms. Based on the gene expression profiles and phenotypic grouping, the parameters were set as follows: minimum gene set size = 5, maximum gene set size = 5000, 1000 resampling permutations. Statistical significance was defined as a p-value < 0.05 and a false discovery rate (FDR) < 0.25.

### 2.7 Statistical analyses

All quantitative data were presented as the mean ± SD. The Mann-Whitney U test or Student’s t-test was used to compare continuous variables between two groups. Multiple comparisons among three or more groups were performed using one-way analysis of variance (ANOVA).The diagnostic value was analyzed using receiver operating characteristic (ROC) curves, with the area under the curve (AUC). The ROC curves were performed using SPSS 17.0 software (SPSS, Chicago, IL, USA). The scatter plots were created with GraphPad Prism 7 software (GraphPad Software, Inc., San Diego, CA, USA). The optimum cut-off value was determined by identifying the maximum value of the Youden’s index (Youden’s index = sensitivity + specificity - 1). All statistical tests were two-sided, and a p-value < 0.05 were considered statistically significant.

## 3 Results

### 3.1 Expression levels of plasma Hsp90α, PLT and platelet-neutrophil ratio (PNR) in patients with PCa and control group

The cohort comprised 144 patients with PCa, and 64 patients with benign prostatic diseases (**Table 1**). Plasma Hsp90α levels were higher in PCa patients than in benign prostatic diseases controls (**Figure 1A**), with the mean ± SD of 102.80 ± 89.77*vs.*62.57 ± 34.82 (ng/mL). In contrast, PLT and PNR levels were lower in PCa patients than benign prostatic diseases (**Figures 1B, C**), with mean ± SD of PLT 213 ± 58.95*vs.*266 ± 70.62(*10^9^/L), PNR 62.48 ± 24.01*vs.*74.33 ± 25.19. Overall, The distribution of plasma Hsp90α, PLT and PNR were significantly difference between PCa patients and benign prostatic diseases controls (*p* < 0.001, *p* = 0.015, *p* = 0.014, respectively, **Figure 1** and **Table 1**).

**Table 1:** Characteristics of prostate cancer and controls.

Characteristic	PCa	benign prostatic diseases	P-value
Age(years)	71.17±8.28	65.27±7.89	0.016
Hsp90α (ng/mL)	102.80±89.77	62.57±34.82	<0.001
Testosterone*(ng/mL)	4.80±1.58	4.80±1.58	/
Neutrophil	4.01±1.57	3.94±1.91	0.590
Lymphocyte	1.57±0.54	1.87±0.53	0.102
NLR	2.82±1.69	2.14±0.71	0.121
PLT	213±58.95	266±70.62	0.014
PLR	152.59±69.13	160.73±95.10	0.840
PNR	62.48±24.01	74.33±25.19	0.015
SII	587.75±401.76	580.32±253.60	0.972
TPSA	419.81±856.26	12.68±10.05	<0.001
FPSA	20.58±21.11	1.54±0.75	<0.001
CEA (ng/mL)#	4.50±10.24	3.18±1.29	/
pT stage(n)			
T1+T2	36		
T3+T4	51		
Tx	57		
N stage(n)			
N0	60		
N1	68		
Nx	16		
M stage(n)			
M0	54		
M1	86		
Mx	3		
Clinical stage (n)			
I+II+III	46		
IV	98		
Gleason Score			
≦6	14		
7	28		
>7	89		
unknown	13		

*PCa 98/144, benign prostatic diseases 33/64

#PCa 73/144, benign prostatic diseases 30/64.

**Figure 1:**
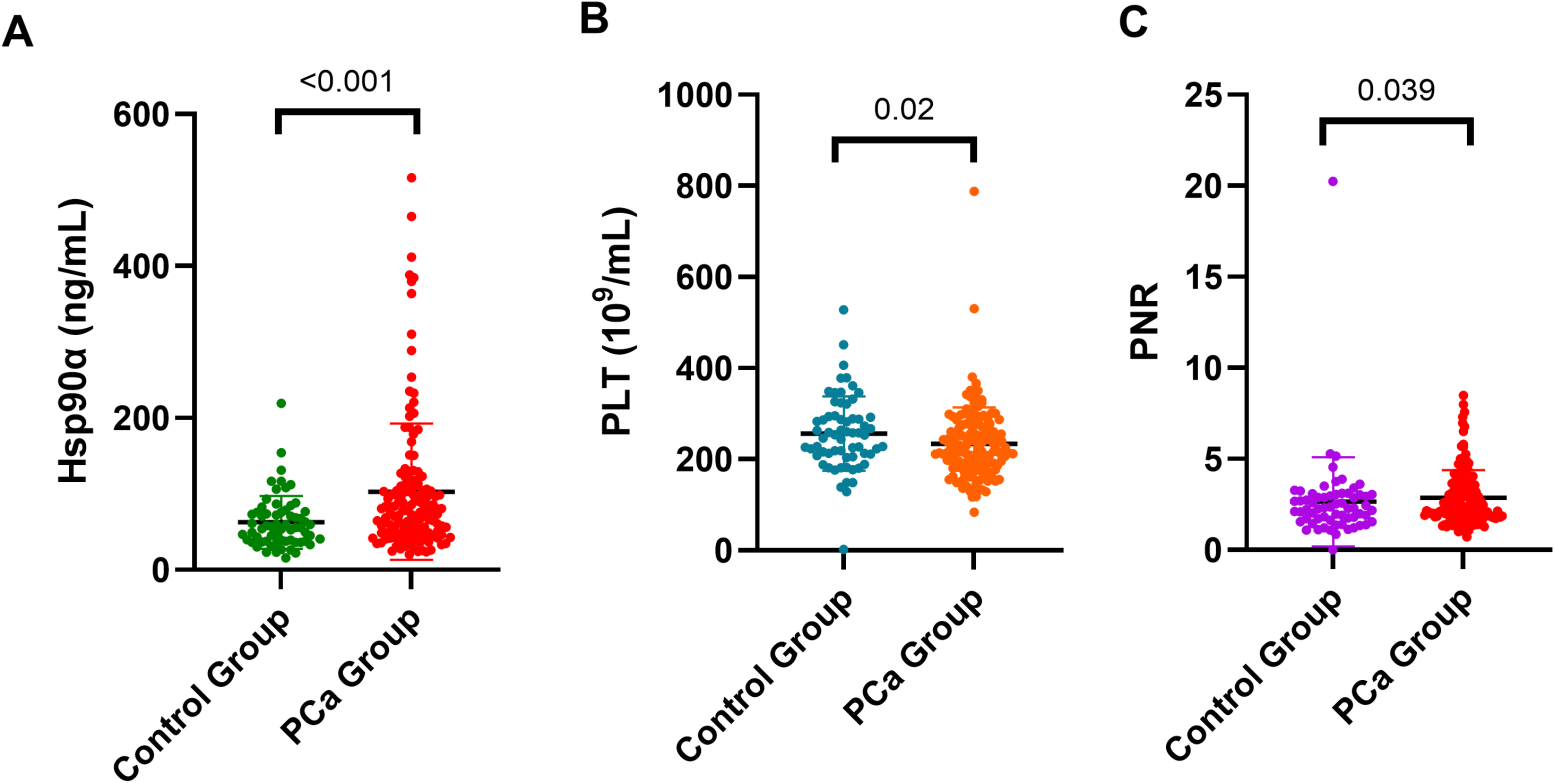
Expression levels of plasma Hsp90α **(A)**, PLT **(B)** and PNR **(C)** in patients with PCa and control group.

### 3.2 Correlation of plasma Hsp90α levels with PSA and inflammatory markers

**Figure 2:**
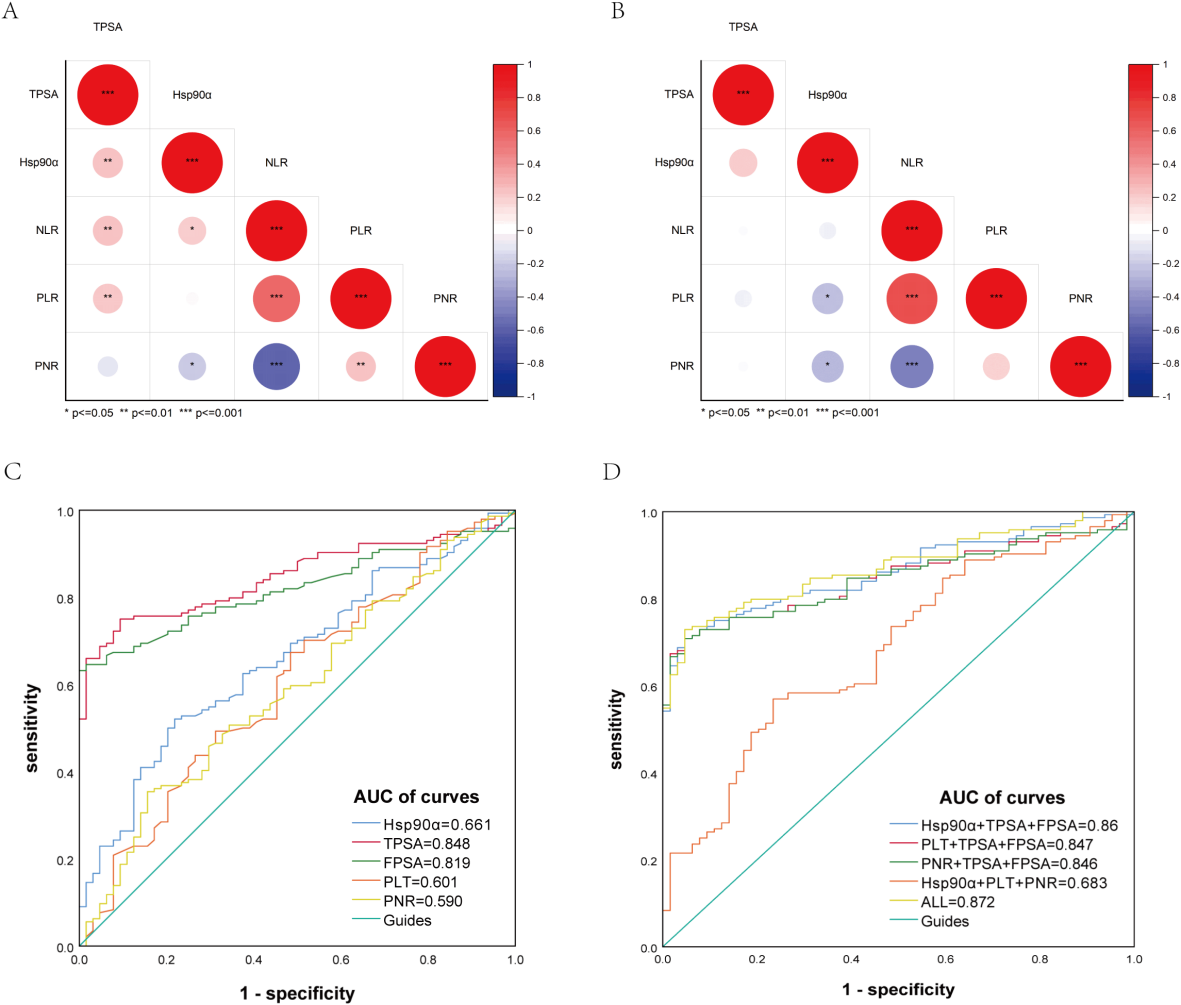
Correlation of plasma Hsp90α levels with PSA, PLT and PNR in patients with PCa **(A)** and control group **(B)**; ROC curves of Hsp90α, PLT, PNR and PSA **(C, D)**.

We performed a correlation analysis of plasma Hsp90α and PSA in PCa (**Figure 1B**), and observed that plasma Hsp90α levels was significantly correlated with PSA (r = 0.25, p < 0.01). Furthermore, by analyzing the correlation between plasma Hsp90α levels and inflammatory markers (NLR, PLR, PNR), we found that plasma Hsp90α levels were closely related to inflammatory markers NLR (r = 0.21, p < 0.05) and PNR (r = -0.20, p < 0.05) (**Figure 2A**). However, we identified no significant correlation of plasma Hsp90α with PSA in benign controls (**Figure 2B**). Taken together, these results indicate that plasma Hsp90α was correlated with PSA and inflammatory markers in PCa.

### 3.3 Association of plasma Hsp90α levels with clinical characteristics in PCa

**Figure 3:**
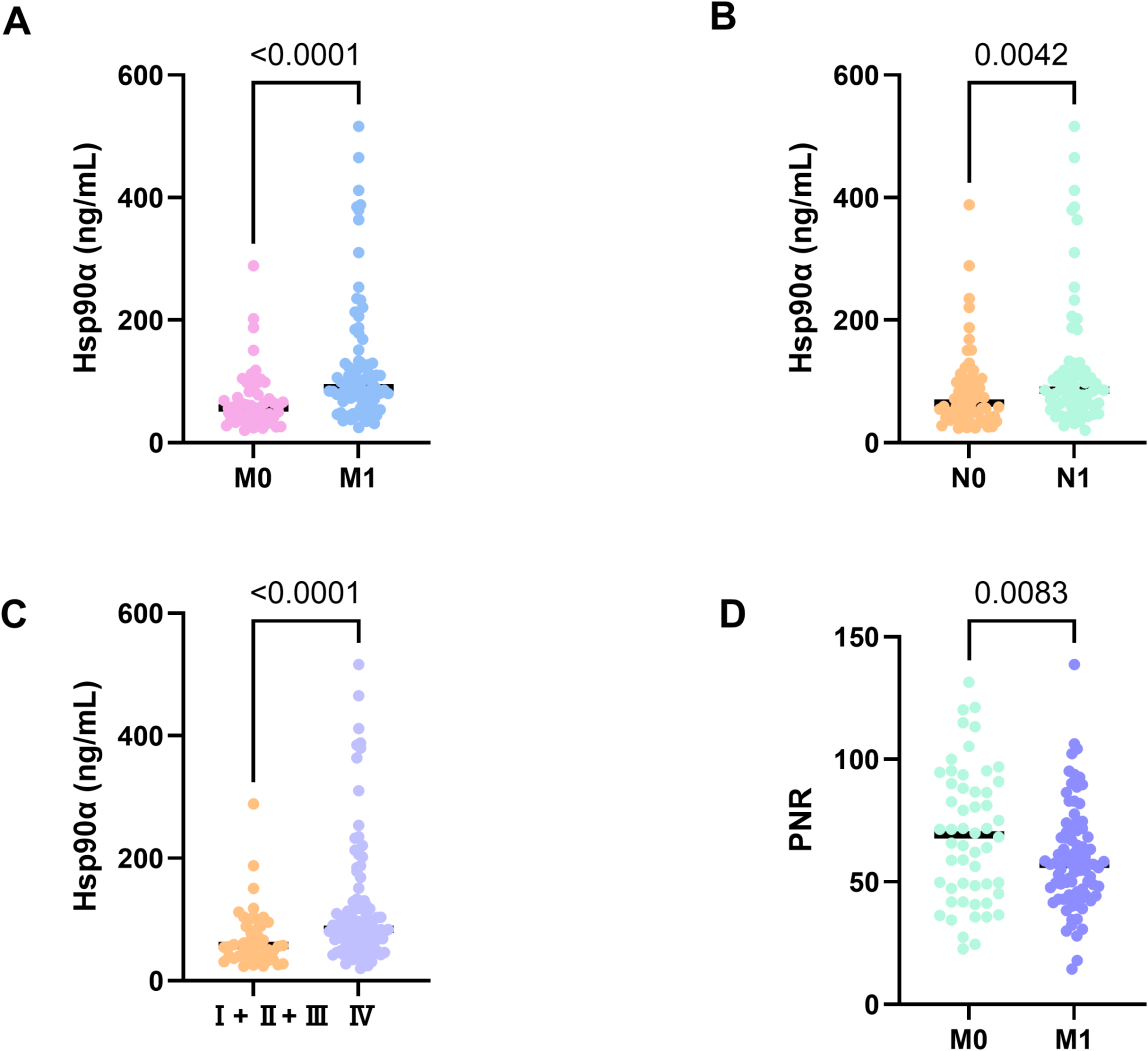
Expression levels of plasma Hsp90α in patients with M stages **(A)**, N stages **(B)** and clinic stages **(C)** in PCa, levels of PNR in patients with M stages **(D)** in PCa.

As shown in **Table 2**, plasma Hsp90α levels were significantly increased in patients with M1 and N1 stage compared to those in M0 and N0 stage (*p* < 0.001, *p* = 0.004, respectively) (**Figure 3A, B**), respectively. Moreover, patients with IV stage presented higher plasma Hsp90α levels compared with those in I+II+III stage (*p* < 0.001) (**Figure 3C**). However, no significant associations were observed in T stage, and Gleason Score (all *p* > 0.05).

**Table 2:** Association of plasma Hsp90α levels with characteristics in prostate cancer.

Variables	Hsp90α levels (ng/mL)	P-value
Age		
≦70yeas	108.83±96.69	0.152
>70yeas	95.87±81.26	
pT stage(n)		
T1+T2	73.89±52.90	0.081
T3+T4	109.05±92.28	
N stage(n)		
N0	85.00±67.32	0.0042
N1	122.79±109.86	
M stage(n)		
M0	69.35±48.68	<0.001
M1	124.86±104.11	
Gleason Score		
≦7	82.10±67.22	0.135
>7	103.77±85.75	
Clinical stage		
I+II+III	70.25±47.86	<0.001
IV	118.08±100.40	

### 3.4 Association of PNR levels with clinical characteristics in PCa

As shown in **Table 3**, PNR levels were significantly decreased in patients with M1 stage compared to those in M0 stage (*p* = 0.005)(**Figure 3D**). However, no significant difference was found regarding the T stage, N stageand, Gleason Score or clinical stage(all *p* > 0.05).

**Table 3:** Association of PLT and PLR levels with characteristics in prostate cancer.

Variables	PLT counts	P-value	PNR	P-value
pT stage(n)				
T1+T2	238.61±76.29	0.733	69.88±24.45	0.291
T3+T4	237.53±59.15		63.98±22.36	
N stage(n)				
N0	233.55±69.20	0.851	66.02±24.19	0.199
N1	229.64±63.41		60.67±23.20	
M stage(n)				
M0	233.2±68.13	0.296	69.19±27.28	0.005
M1	232.73±86.99		59.79±20.87	
Gleason Score				
≦7	238.24±73.13	0.681	66.59±24.75	0.860
>7	233.18±85.99		64.09±24.65	
Clinical stage				
I+II+III	233.04±71.98	0.571	67.63±26.14	0.109
IV	233.78±83.91		62.21±23.26	

### 3.5 Diagnostic value of plasma Hsp90α, PLT Counts and PNR levels in PCa

Using Prostatitis and BPH as controls, we found that plasma Hsp90α has a moderate diagnostic value in gastric cancer with the cut-off value as 76.85 (ng/mL), with the sensitivity and specificity of 0.521, 0.781, and the AUC of 0.661, respectively. Meanwhile, PLT Counts and PNR levels has a moderate diagnostic value in gastric cancer with the cut-off value as 252(*10^9^/L), 50.34, with the sensitivity and specificity as 0.674, 0.516 and 0.354,0.844, and the AUC of 0.601 and 0.590, respectively, (**Figure 2C**). we found that TPSA and FPSA have high diagnostic value in PCa just as the previous author described. Moreover, based on the multivariate regression analysis results, we determined that the diagnostic value of increased moderately, with the AUC of 0.872,When Hsp90α, PLT Counts, PNR levels combined with TPSA and FPSA (**Figure 2D**).

### 3.6 Analysis of HSP90AB1 gene expression in TCGA database

**Figure 4:**
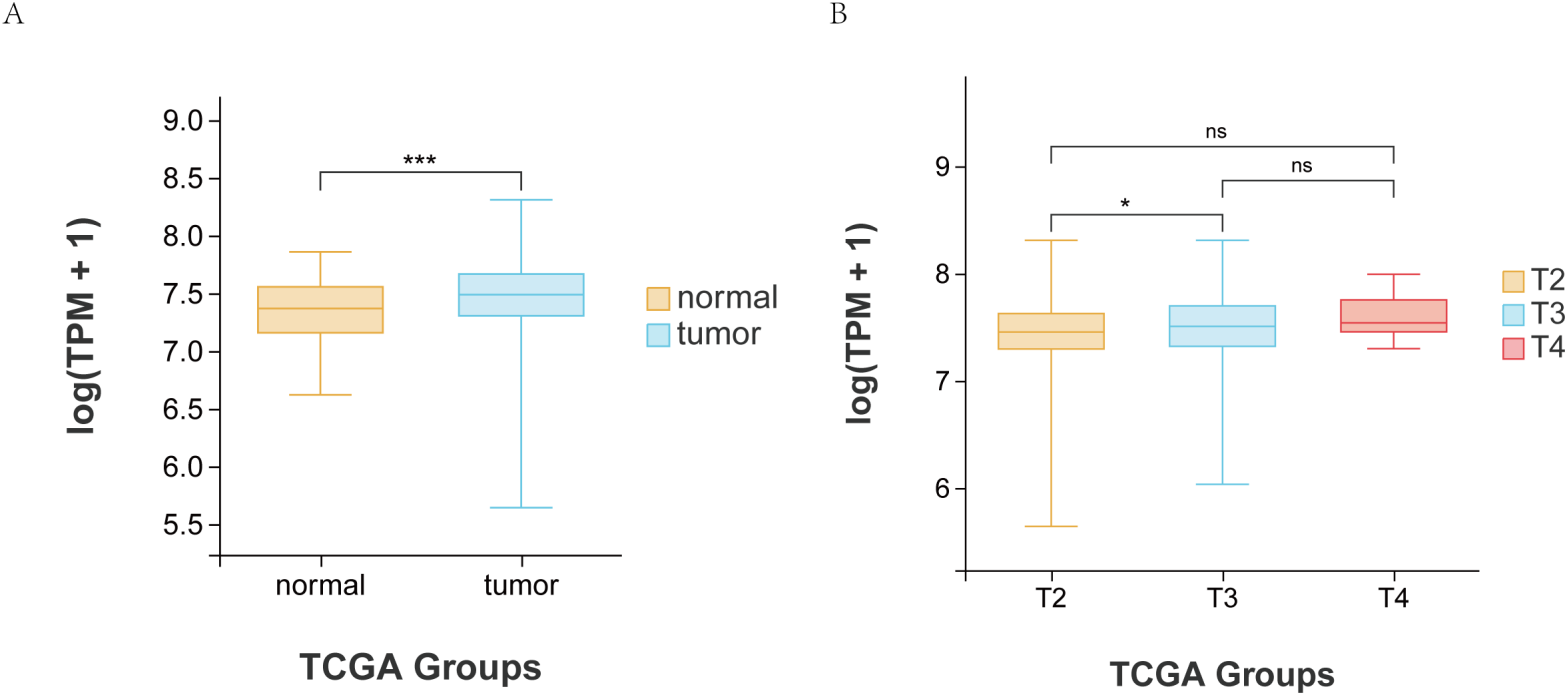
The levels of HSP90AB1 RNA-seq in PCa and adjacent normal tissues from TCGA database **(A)**, The levels of HSP90AB1 RNA-seq in PCa T stages from TCGA database **(B)**. ***:p<0.001, ns: Not Significant.

Through the retrieval and analysis of the TCGA database, we observed that the expression of HSP90AB1 gene in PCa tissue was significantly higher than adjacent non-cancer tissues (*p* < 0.001; **Figure 4A**). Furthermore, HSP90AB1 expression was significantly increased in patients with T3 stage compared to those in T2 stage (*p* = 0.014). Notably, no T1 stage patients were available for analysis within the TCGA-PRAD cohort, a reflection of the dataset’s composition which focuses on higher-stage primary tumors. However, no significant differences were found between T2 *vs.* T4, T3 *vs.* T4, N0 *vs.* N1, or M0 *vs.* M1 (all *p*>0.05, **Figure 4B** and **Table 4**).

**Table 4:** Characteristics of Hsp90AB1 in prostate cancer using TCGA data.

Compare	statistical significance	P.significance
normal(n = 52)-vs-tumor(n = 497)	0.0001	***
T2(n = 185)-vs-T3(n = 293)	0.0141	*
T2(n = 185)-vs-T4(n = 10)	0.1044	ns
T3(n = 293)-vs-T4(n = 10)	0.3020	ns
N0(n = 343)-vs-N1(n = 79)	0.0855	ns
M0(n = 453)-vs-M1(n = 3)	0.6696	ns

*** : p < 0.001, *: p < 0.05, ns : not significant

### 3.7 Bioinformatics analyses showed the biological processes involved and major target gene pathways affected

**Figure 5:**
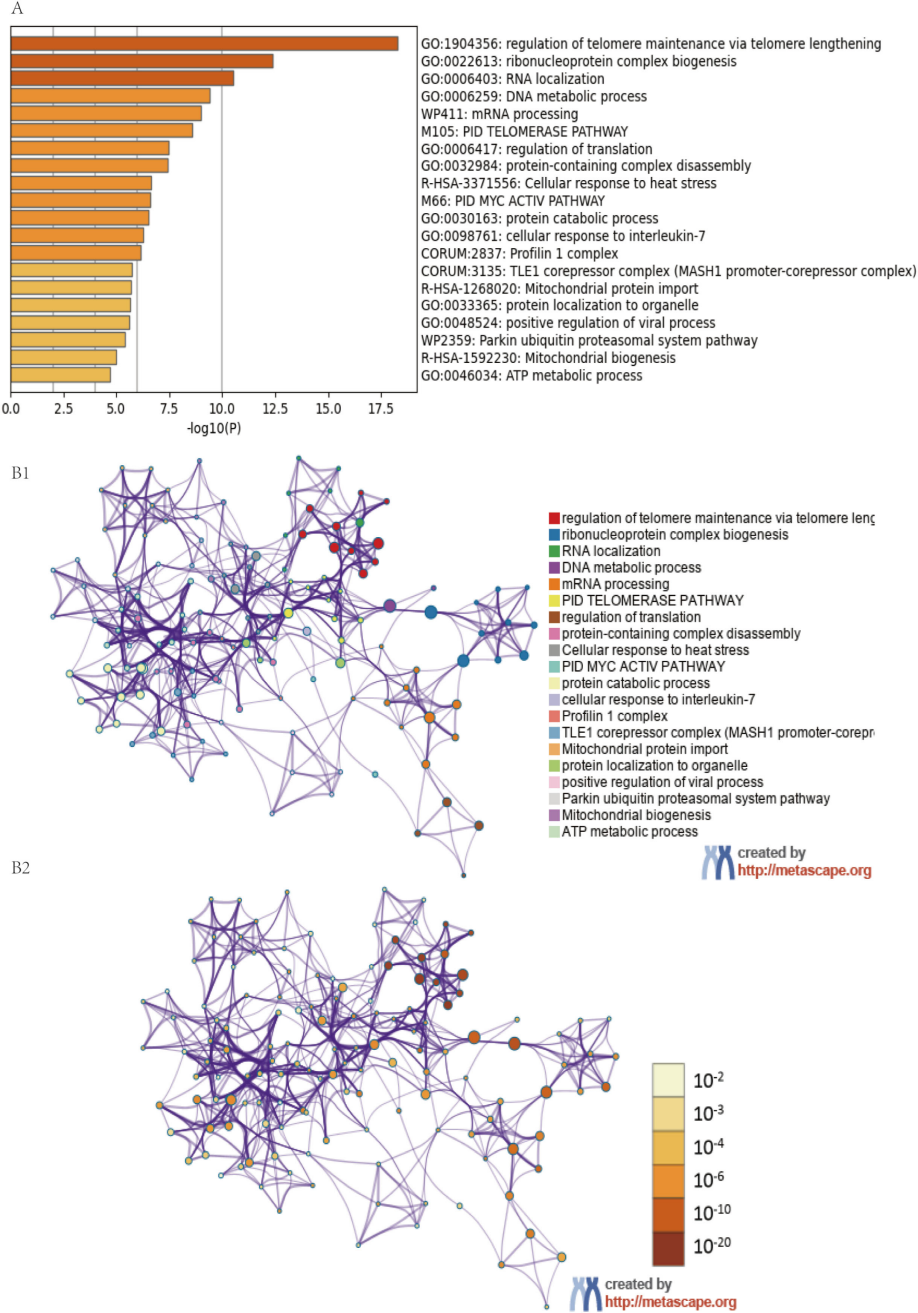
Pathway enrichment showing Hsp90α might impact biological pathways by metascape. **(A)** Functional enrichment analysis bar plot; **(B)** Network visualization of enrichment results.

**Figure 6:**
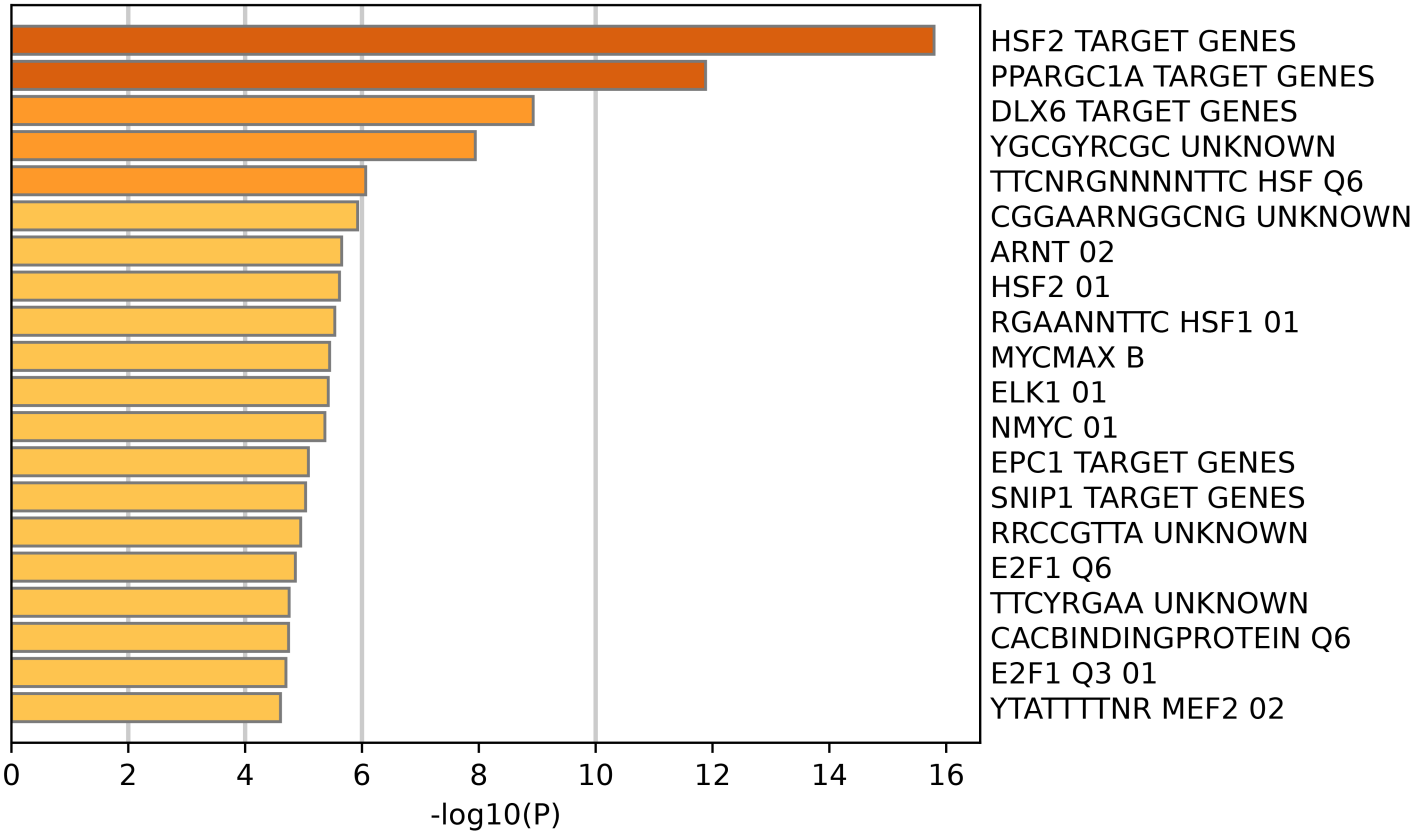
Transcript factors that might regulate Hsp90 expression.

We explored the biological pathways and processes associated with HSP90AB1, the main coding gene of Hsp90, through functional enrichment analysis. First, Pearson’s correlation coefficients between HSP90AB1 expression and other mRNAs were calculated based on the TCGA dataset, and the top 100 genes with the highest correlation values (Pearson’s correlation ≥ 0.3, P < 0.0001) were selected. Pathway and process enrichment analyses results showed that pathways affected by HSP90AB1 were primarily enriched in telomere maintenance via telomere lengthening, ribonucleoprotein complex biogenesis, RNA localization, DNA metabolic process and mRNA processing (**Figure 5**). To further analyze the related target gene pathways regulated by HSP90AB1, enrichment analysis of transcription factor targets was conducted, indicating that the target pathways of the included genes were mainly enriched in HSF2 target genes, PRAGC1A target genes, and DLX6 target genes (**Figure 6**).

### 3.8 Hsp90α-associated biological pathways

**Figure 7:**
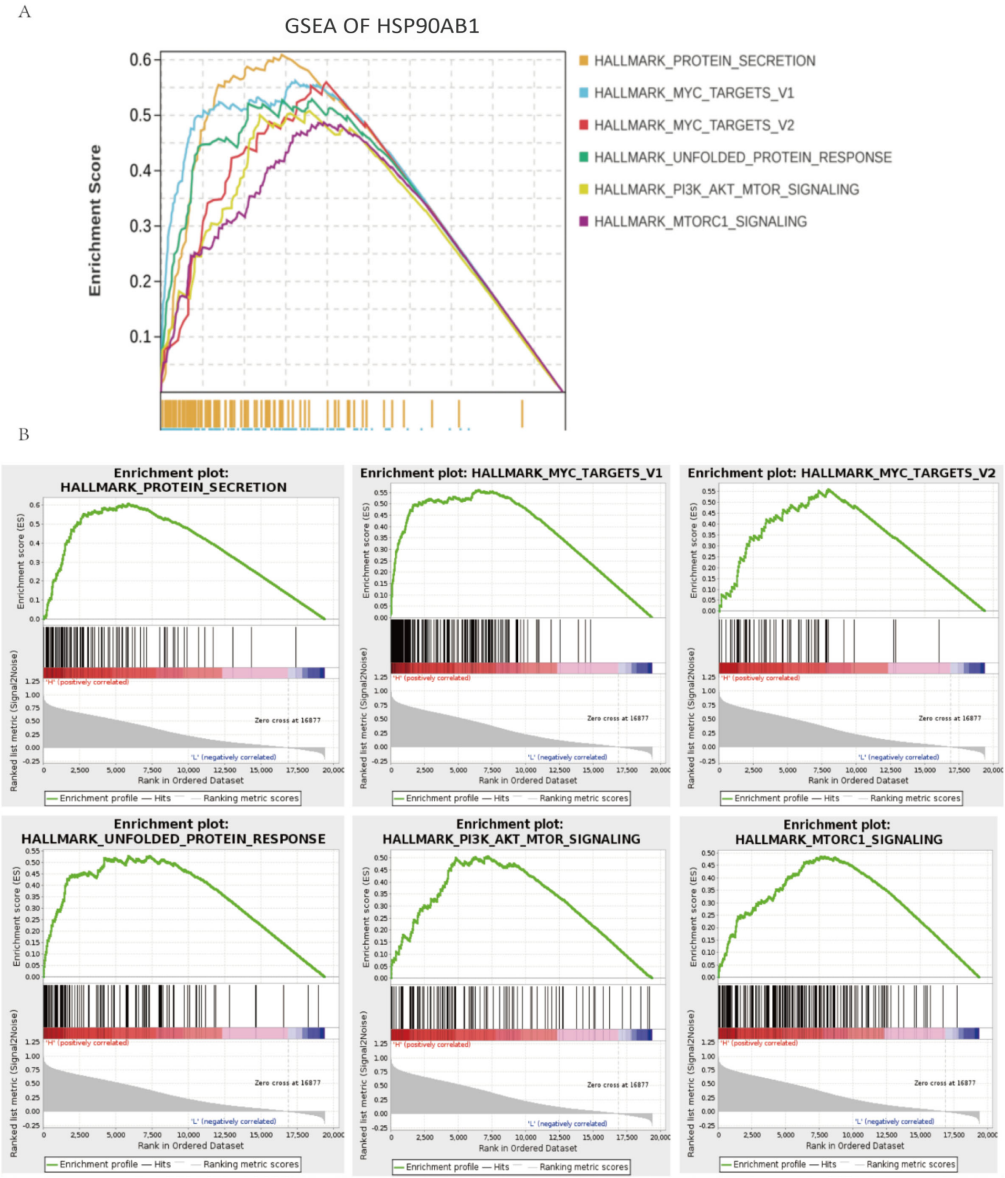
Hsp90α positively correlated pathways details by GSEA. **(A)** Enrichment scores of multiple hallmark gene sets. **(B)** Detailed view of enriched pathways.

To deeply investigate the molecular mechanisms by which Hsp90α influences PCa, we employed the GSEA algorithm to identify significantly altered signaling pathways. Specifically, in the GSEA, by comparing 50 hallmark gene sets, we found a significant association between HSP90AB1 expression and the activation of multiple biological processes related to protein secretion and cell cycle regulation, which include MYC targets, Unfolded Protein Response (UPR) signaling, PI3K/AKT/mTOR signaling, and mTORC1 signaling (**Figure 7**).

## 4 Discussion

We report for the first time that plasma Hsp90α levels are significantly elevated in PCa patients compared to those with benign prostatic diseases. Furthermore, Hsp90α levels exhibited significant positive correlations with metastasis (M stage), lymph node involvement (N stage), and clinical stage, strongly suggesting its involvement in PCa progression. Concurrently, PCa patients showed significantly lower levels of PLT and PNR. Notably, PNR was found to be negatively correlated with distant metastasis (M stage). ROC curve analysis demonstrated that plasma Hsp90α, PLT, and PNR each possess moderate diagnostic efficacy for distinguishing PCa from benign prostatic diseases, supporting their potential as supplementary diagnostic tools to the cornerstone biomarker PSA. Additionally, through bioinformatics analyses—including functional enrichment and gene set enrichment analysis (GSEA)—we identified key signaling pathways and biological processes regulated by Hsp90α in PCa pathogenesis and development. Hsp90α and PNR, as novel biomarkers, demonstrate clear potential for clinical application in the auxiliary diagnosis, risk stratification, and disease progression monitoring of PCa. Their laboratory detection is technically feasible and cost-effective, making them particularly suitable for complementary use alongside PSA.

Hsp90α, an evolutionarily conserved and essential molecular chaperone, is induced by heat shock or stress and secreted into the extracellular space by cancer cells (21, 22). Accumulating evidence indicates that Hsp90α plays a critical role in regulating the conformation, stability, and function of oncogenic proteins, and is involved in processes such as cell proliferation, apoptosis, cell cycle progression, migration, and invasion (9, 16, 23). Clinically, studies have demonstrated that plasma Hsp90α levels are significantly higher in patients with lung cancer than in healthy controls, supporting its utility as a diagnostic biomarker (5). Our study not only confirmed significantly elevated plasma Hsp90α levels in PCa patients but also validated the upregulation of its encoding gene HSP90AB1 in prostate tumor tissues using the TCGA database, providing molecular-level support for its biological significance in PCa. Importantly, plasma Hsp90α levels showed a significant positive correlation with PSA and associations with inflammatory markers (NLR and PNR), suggesting its potential role as a diagnostic and monitoring biomarker linking inflammation to PCa development.

However, no significant differences in plasma Hsp90α levels were observed across different primary tumor extents (T stages) in our cohort. This may be attributed to limited systemic inflammatory responses elicited by localized tumor progression in early stages, resulting in a weaker correlation between circulating Hsp90α and T stage. Study limitations, including the lack of precise T-stage data for 57 metastatic patients who only underwent biopsy only, might also be a contributing factor. In contrast, plasma Hsp90α levels were significantly higher in M1/N1 patients compared to M0/N0 patients, highlighting its potential role in advanced/metastatic PCa. It is noteworthy that TCGA data analysis yielded partially inconsistent results, indicating the need for larger-scale studies to definitively clarify the relationship between Hsp90α expression and clinicopathological features. Despite the crucial role of Hsp90α in tumorigenesis and progression, the clinical efficacy of Hsp90 inhibitors remains suboptimal (5, 6, 9, 16), emphasizing the need for deeper investigation into its specific regulatory mechanisms.

To further explore these mechanisms, we identified 100 genes positively correlated with HSP90AB1 expression in PCa. Functional enrichment analysis was performed via bioinformatics approaches to explore the biological pathways and processes associated with HSP90AB1. The results showed that these 100 genes were primarily enriched in the following biological processes: energy metabolism and mitochondrial homeostasis, protein quality control network, gene expression regulatory cascade, stress response, and immune regulation. Additionally, enrichment analysis of transcription factor target genes indicated that the target pathways of the included genes were mainly enriched in heat shock factor 2 (HSF2) target genes, PRAGC1A target genes, and DLX6 target genes. Previous studies have demonstrated that HSF2 might be involved in the onset of HSPs and regulate their expression (24), and DLX6 promotes cell proliferation and suppresses apoptosis in oral cancer cells (25). GSEA results reinforced these findings, showing that high HSP90AB1 expression levels were significantly associated with the activation of multiple pro-oncogenic pathways, including: the MYC target gene pathway, unfolded protein response (UPR), PI3K/AKT/mTOR signaling pathway, mTORC1 signaling pathway, and protein secretion-related pathways. Together, these results systematically delineate a complex network through which Hsp90α promotes PCa initiation and progression by orchestrating energy metabolism, protein homeostasis maintenance, activation of key pro-proliferative signaling pathways, and immune microenvironment modulation, providing novel insights into its biological functions and potential targeted therapeutic strategiese (26).

PLT express multiple functional Toll-like receptors (TLR) and can also secrete both pro-infammatory and anti-infammatory cytokines (how platelets regulate when to induce or suppress infammation remains to be studied), thereby modulating immune system function (27). Several studies have focused on the effect of platelet-leukocyte interplay on tumor cell extravasation and survival. PLT and granulocytes together form the premetastatic niche for injected colon carcinoma cells (28). The value of inflammatory markers (e.g., NLR, PLR, SII) in PCa diagnosis, prognosis assessment, and treatment response prediction has been extensively studied (29–31). For instance, elevated NLR is associated with an increased risk of PCa (14), specific marker combinations (e.g., prostate volume index (PVI) and NLR) can improve the early detection rate of PCa in the diagnostic gray zone (31), and inflammatory indices are significantly correlated with poorer overall survival (OS) in patients with metastatic castration-resistant prostate cancer (mCRPC) (30). Our study observed significantly lower PLT and PNR levels in PCa patients compared to benign prostatic diseases patients. Crucially, PNR is reported here for the first time to possess moderate diagnostic value for PCa and is negatively correlated with distant metastasis. This finding expands the scope of research on inflammatory markers in PCa, which has predominantly focused on NLR, PLR, and SII (14, 15, 30). The pivotal role of PSA in prostate cancer diagnosis is undeniable. However, inflammatory markers offer greater universality and cost-effectiveness in testing. This makes them highly attractive for potential clinical application and broader dissemination. Moreover, studies have demonstrated the predictive diagnostic value of the mean platelet volume to platelet count ratio and neutrophil to lymphocyte ratios specifically within the diagnostic gray zone of prostate cancer, where tPSA levels range between 4 to 10 ng/mL (31). The underlying biological basis may involve neutrophils promoting tumor development by mediating immunosuppression, angiogenesis, and pre-metastatic niche formation (32, 33), while platelets play a key role in facilitating cancer metastasis (28). Notably, no significant correlation was observed between NLR and PCa in our study, consistent with some previous reports (34). Similarly, PLR did not show statistically significant differences in this cohort, contrasting with findings from other studies (35). This suggests that heterogeneity in population, geographic region, or research methodology may contribute to variations in the value of inflammatory markers, factors that future research should carefully consider.

This study has several limitations that should be considered. First, its retrospective nature inherently carries a potential risk of selection bias. Furthermore, the sample size, particularly of early-stage patients, remains relatively limited compared to those with advanced disease, which may affect the statistical balance and generalizability of some findings. We also acknowledge that while we sought to control for key confounders, unmeasured variables such as specific comorbidities or concomitant medications could potentially influence plasma Hsp90α levels. Additionally, precise T-stage information was unavailable for a subset of cases, specifically those with metastatic disease diagnosed only by biopsy. Consequently, the conclusions of this study, particularly the novel associations involving Hsp90α and PNR, necessitate further validation in larger-scale, prospectively designed cohorts. Future research should be directed toward: 1) conducting in-depth validation at the tissue and molecular levels using a prospective cohort with a comprehensive biobank; 2) elucidating the specific signaling pathways (e.g., those related to HSF2 and DLX6) through which Hsp90α operates in prostate cancer; 3) uncovering the biological mechanisms underpinning PNR’s role in metastasis; and 4) rigorously evaluating and refining diagnostic and prognostic models that integrate Hsp90α, PSA, and novel inflammatory markers such as PNR.

## 5 Conclusion

In summary, this study establishes the potential clinical utility of plasma Hsp90α as an auxiliary diagnostic biomarker for PCa, particularly in advanced or metastatic disease. Furthermore, we are the first to report the diagnostic and distant metastasis risk assessment potential of the PNR in PCa. Importantly, a diagnostic model integrating Hsp90α and PNR with the cornerstone biomarker PSA demonstrated superior diagnostic performance compared to the use of PSA alone, highlighting their complementary role. Through integrated bioinformatics analyses, we have elucidated the molecular mechanisms by which Hsp90α drives PCa progression, revealing its involvement in a complex regulatory network governing energy metabolism, protein homeostasis, key pro-proliferative signaling pathways (e.g., PI3K/AKT/mTOR, MYC), and immune microenvironment modulation. These findings provide novel mechanistic insights into PCa pathophysiology and lay a foundation for the future development of diagnostic strategies and targeted therapies focused on Hsp90α or its associated pathways.

## Data Availability

The raw data supporting the conclusions of this article will be made available by the authors, without undue reservation.

## References

[1] Adamaki M Zoumpourlis V . Prostate Cancer Biomarkers: From diagnosis to prognosis and precision-guided therapeutics. Pharmacol Ther. (2021) 228. doi: 10.1016/j.pharmthera.2021.107932, PMID: 34174272

[2] Bray F Laversanne M Sung H Ferlay J Siegel RL Soerjomataram I . Global cancer statistics 2022: GLOBOCAN estimates of incidence and mortality worldwide for 36 cancers in 185 countries. CA: A Cancer J Clin. (2024) 74:229–63. doi: 10.3322/caac.21834, PMID: 38572751

[3] Siegel RL Giaquinto AN Jemal A . Cancer statistics, 2024. CA: A Cancer J Clin. (2024) 74:12–49. doi: 10.3322/caac.21820, PMID: 38230766

[4] Hoter A El-Sabban ME Naim HY . The HSP90 family: structure, regulation, function, and implications in health and disease. Int J Mol Sci. (2018) 19. doi: 10.3390/ijms19092560, PMID: 30158430 PMC6164434

[5] Shi Y Liu X Lou J Han X Zhang L Wang Q . Plasma levels of heat shock protein 90 alpha associated with lung cancer development and treatment responses. Clin Cancer Res. (2014) 20:6016–22. doi: 10.1158/1078-0432.CCR-14-0174, PMID: 25316816

[6] Fu Y Xu X Huang D Cui D Liu L Liu J . Plasma heat shock protein 90alpha as a biomarker for the diagnosis of liver cancer: an official, large-scale, and multicenter clinical trial. EBioMedicine. (2017) 24:56–63. doi: 10.1016/j.ebiom.2017.09.007, PMID: 28939487 PMC5652007

[7] Cheng Q Chang JT Geradts J Neckers LM Haystead T Spector NL . Amplification and high-level expression of heat shock protein 90 marks aggressive phenotypes of human epidermal growth factor receptor 2 negative breast cancer. Breast Cancer Res. (2012) 14. doi: 10.1186/bcr3168, PMID: 22510516 PMC3446397

[8] Hance MW DK Gopal U Bohonowych JE Jezierska-Drutel A Neumann CA Liu H . Secreted Hsp90 is a novel regulator of the epithelial to mesenchymal transition (EMT) in prostate cancer. J Biol Chem. (2012) 287:37732–44. doi: 10.1074/jbc.M112.389015, PMID: 22989880 PMC3488049

[9] Duan C LK Pan X Wei Z Xiao L . Hsp90 is a potential risk factor for ovarian cancer prognosis: an evidence of a Chinese clinical center. BMC Can. (2023) 23:489. doi: 10.1186/s12885-023-10929-9, PMID: 37259027 PMC10230804

[10] Prodromou C . Mechanisms of hsp90 regulation. Biochem J. (2016) 473:2439–52. doi: 10.1042/BCJ20160005, PMID: 27515256 PMC4980810

[11] Colotta F Allavena P Sica A Garlanda C Mantovani A . Cancer-related inflammation, the seventh hallmark of cancer: links to genetic instability. Carcinogenesis. (2009) 30:1073–81. doi: 10.1093/carcin/bgp127, PMID: 19468060

[12] Mungan İ Dicle ÇB Bektaş Ş Sarı S Yamanyar S Çavuş M . Does the preoperative platelet-to-lymphocyte ratio and neutrophil-to-lymphocyte ratio predict morbidity after gastrectomy for gastric cancer? Militar Med Res. (2020) 7. doi: 10.1186/s40779-020-00234-y, PMID: 32111261 PMC7049207

[13] Li J Zheng J Wang P Lv D . Prognostic significance of hemoglobin, albumin, lymphocyte and platelet score in solid tumors: a pooled study. Front Immunol. (2024) 15. doi: 10.3389/fimmu.2024.1483855, PMID: 39744624 PMC11688271

[14] Wang L Li X Liu M Zhou H Shao J . Association between monocyte-to-lymphocyte ratio and prostate cancer in the U.S. population: a population-based study. Front Cell Dev Biol. (2024) 12. doi: 10.3389/fcell.2024.1372731, PMID: 38645410 PMC11026607

[15] He R Ye Y Zhu Q Xie C . Systemic immune-inflammation index is associated with high risk for prostate cancer among the U.S. elderly: Evidence from NHANES 2001-2010. Front Oncol. (2024) 14. doi: 10.3389/fonc.2024.1441271, PMID: 39376981 PMC11456397

[16] Wei W Liu M Ning S Wei J Zhong J Li J . Diagnostic value of plasma HSP90α levels for detection of hepatocellular carcinoma. BMC Can. (2020) 20. doi: 10.1186/s12885-019-6489-0, PMID: 31898536 PMC6941289

[17] Weinstein JN Collisson EA Mills GB Shaw KRM Ozenberger BA Ellrott K . The Cancer Genome Atlas Pan-Cancer analysis project. Nat Genet. (2013) 45:1113–20. doi: 10.1038/ng.2764, PMID: 24071849 PMC3919969

[18] Tripathi S Pohl Marie O Zhou Y Rodriguez-Frandsen A Wang G Stein David A . Meta- and orthogonal integration of influenza “OMICs” Data defines a role for UBR4 in virus budding. Cell Host Microbe. (2015) 18:723–35. doi: 10.1016/j.chom.2015.11.002, PMID: 26651948 PMC4829074

[19] Subramanian A Tamayo P Mootha VK Mukherjee S Ebert BL Gillette MA . Gene set enrichment analysis: a knowledge-based approach for interpreting genome-wide expression profiles. Proc Natl Acad Sci. (2005) 102:15545–50. doi: 10.1073/pnas.0506580102, PMID: 16199517 PMC1239896

[20] Liberzon A Birger C Thorvaldsdóttir H Ghandi M Mesirov Jill P Tamayo P . The molecular signatures database hallmark gene set collection. Cell Syst. (2015) 1:417–25. doi: 10.1016/j.cels.2015.12.004, PMID: 26771021 PMC4707969

[21] Eustace BK Sakurai T Stewart JK Yimlamai D Unger C Zehetmeier C . Functional proteomic screens reveal an essential extracellular role for hsp90α in cancer cell invasiveness. Nat Cell Biol. (2004) 6:507–14. doi: 10.1038/ncb1131, PMID: 15146192

[22] Frydman J . Folding of newly translated proteins *in vivo*: the role of molecular chaperones. Annu Rev Biochem. (2001) 70:603–47. doi: 10.1146/annurev.biochem.70.1.603, PMID: 11395418

[23] Liang X-Q Li K-Z Li Z Xie M-Z Tang Y-P Du J-B . Diagnostic and prognostic value of plasma heat shock protein 90alpha in gastric cancer. Int Immunopharmacol. (2021) 90. doi: 10.1016/j.intimp.2020.107145, PMID: 33162344

[24] Loones MT Mezger MR,V Morange M . Affiliations. HSP gene expression and HSF2 in mouse development. Cell Mol Life Sci. (1997) 53:179–90. doi: 10.1007/PL00000590, PMID: 9118006 PMC11147342

[25] Liang J Liu J Deng Z Liu Z Liang L . DLX6 promotes cell proliferation and survival in oral squamous cell carcinoma. Oral Dis. (2020) 28:87–96. doi: 10.1111/odi.13728, PMID: 33215805

[26] Park H-K Lee J-E Lim J Kang BH . Mitochondrial Hsp90s suppress calcium-mediated stress signals propagating from mitochondria to the ER in cancer cells. Mol Cancer. (2014) 13. doi: 10.1186/1476-4598-13-148, PMID: 24924916 PMC4070406

[27] Holinstat M . Normal platelet function. Cancer Metast Rev. (2017) 36:195–8. doi: 10.1007/s10555-017-9677-x, PMID: 28667366 PMC5709181

[28] Zhou L Zhang Z Tian Y Li Z Liu Z Zhu S . The critical role of platelet in cancer progression and metastasis. Eur J Med Res. (2023) 28. doi: 10.1186/s40001-023-01342-w, PMID: 37770941 PMC10537080

[29] Morais M Fonseca T MaChado-Neves R Honavar M Coelho AR Lopes J . Prognostic value of neutrophil-to-lymphocyte ratio (NLR) and platelet-neutrophil (PN) index in locally advanced rectal cancer patients: a retrospective cohort study. Ann Med Surg. (2024) 86:2474–80. doi: 10.1097/MS9.0000000000001297, PMID: 38694305 PMC11060258

[30] Guan Y Xiong H Feng Y Liao G Tong T Pang J . Revealing the prognostic landscape of neutrophil-to-lymphocyte ratio and platelet-to-lymphocyte ratio in metastatic castration-resistant prostate cancer patients treated with abiraterone or enzalutamide: a meta-analysis. Prostate Cancer Prostatic Dis. (2020) 23:220–31. doi: 10.1038/s41391-020-0209-3, PMID: 32034294

[31] Yi X Li J Li Y Huang T Xiong B Zhang F . Predictive diagnostic value of mean platelet volume to platelet count and neutrophil to lymphocyte ratios in the gray zone of prostate cancer with tPSA between 4 to 10 ng/mL. Front Oncol. (2024) 14. doi: 10.3389/fonc.2024.1454124, PMID: 39469646 PMC11513254

[32] Coffelt SB Kersten K Doornebal CW Weiden J Vrijland K Hau C-S . IL-17-producing γδ T cells and neutrophils conspire to promote breast cancer metastasis. Nature. (2015) 522:345–8. doi: 10.1038/nature14282, PMID: 25822788 PMC4475637

[33] Masuda H Mikami K Otsuka K Hou K Suyama T Araki K . Validation of the effectiveness of neutrophil-to-lymphocyte ratio (NLR) as a predictive factor in patients undergoing prostate biopsy with prostate specific antigen (PSA) between 4.0 and 10.0 ng/ml. In Vivo. (2021) 35:1641–6. doi: 10.21873/invivo.12422, PMID: 33910847 PMC8193316

[34] Kaynar M Yildirim ME Gul M Kilic O Ceylan K Goktas S . Benign prostatic hyperplasia and prostate cancer differentiation via platelet to lymphocyte ratio. Cancer Biomark. (2015) 15:317–23. doi: 10.3233/CBM-150458, PMID: 25586096 PMC12964681

[35] Adhyatma KP Warli SM . Diagnostic value of platelet–to-lymphocyte ratio in prostate cancer. Open Access Macedonian J Med Sci. (2019) 7:1093–6. doi: 10.3889/oamjms.2019.252, PMID: 31049087 PMC6490494

